# Measurement of the Coherent Neutron Scattering Length of ^3^*He*

**DOI:** 10.6028/jres.110.032

**Published:** 2005-06-01

**Authors:** W. Ketter, W. Heil, G. Badurek, M. Baron, R. Loidl, H. Rauch

**Affiliations:** Institut für Physik, Universität Mainz, Germany; Atominstitut der Österreichischen Universitäten, Wien, Austria

**Keywords:** elastic neutron scattering, few body systems, neutron interferometry, properties of the ^3^He nucleus, real part of the coherent scattering length

## Abstract

By means of neutron interferometry the s-wave neutron scattering length of the ^3^He nucleus was re-measured at the Institut Laue-Langevin (ILL). Using a skew symmetrical perfect crystal Si-interferometer and a linear twin chamber cell, false phase shifts due to sample misalignment were reduced to a negligible level. Simulation calculations suggest an asymmetrically alternating measuring sequence in order to compensate for systematic errors caused by thermal phase drifts. There is evidence in the experiment’s data that this procedure is indeed effective. The neutron refractive index in terms of Sears’ exact expression for the scattering amplitude has been analyzed in order to evaluate the measured phase shifts. The result of our measurement, *b′*_c_ = (6.000 ± 0.009) fm, shows a deviation towards a greater value compared to the presently accepted value of *b′*_c_ = (5.74 ± 0.07) fm, confirming the observation of the partner experiment at NIST. On the other hand, the results of both precision measurements at NIST and ILL exhibit a serious 12σ (12 standard uncertainties) deviation, the reason for which is not clear yet.

## 1. Introduction

The scattering length *a* is defined as the zero-energy limit of the scattering amplitude *f*. Owing to the spin dependence of the underlying strong interaction, the neutron scattering length must in general also be spin dependent. The spin independent part of the quantity is referred to as the coherent scattering length *a*_c_. In order to expand the scattering formalism to absorption, the scattering length is made complex, *a* = *a′* − *ia″*. For a homogeneous, isotropic, monatomic and dilute gas sample of particle number density *ρ*, neutron scattering can be described by the so-called coherent wave that scatters off a macroscopic optical potential. Since this effective potential is weak in general, one can define a neutron refractive index and treat the scattering problem similarly to geometrical light optics. When describing the scattering off a nucleus of mass number *A* in the laboratory system, one often includes the kine-matical transformation from the centre of mass system by defining the bound scattering length $b=\frac{A+1}{A}a$. Sears [1] has given an exact expression of the neutron refractive index in terms of the scattering amplitude. Taking into account that the scattering amplitude *f* and the scattering length *a* are proportional only in the zero-energy limit, but that in general higher-order terms in the neutron wave number *k* contribute, one arrives at the following expression for the real (nonabsorptive) part of the refractive index in terms of the bound coherent scattering length *b*_c_:

$$n\prime =1-\frac{2\pi }{k^{2}}\rho b_{c}^{\prime }[1-2kb_{c}^{\prime \prime }+O(k^{2})].$$ (1)

Since scattering lengths are of the order of a few femtometers, the second term in the bracket is of the order of 10^−4^ for thermal neutrons and the term in *k*^2^ can safely be ignored for our purposes.

In the following section, we describe an interferometric experiment that measures the phase shift

Δφ=(n′−1)kd (2)

that the partial wave function of a neutron of kinetic energy *E*_k_ = *ħ*^2^*k*^2^/2*m* experiences when passing through a sample of thickness *d*.

## 2. Experiment

The experiment was performed in May 2002 at the S18 CRG facility at ILL using the skew symmetrically shaped perfect crystal interferometer IFM4. The setup is essentially the same as described in [2], the most important changes being a massive water-cooled Helmholtz coil of 50 cm diameter with the interferometer at its centre and two layers of Mylar^1^ foil between the coils’ body and the interferometer. The coil was initially installed for polarized interferometry but turned out to stabilize the instrument’s temperature considerably when operated.

On their optical path through the interferometer, the partial beams gather a phase difference ∆*φ* that causes modulations of the detectable intensity behind the instrument:

$$I_{0,H}\propto 1+\cos\Delta \varphi .$$ (3)

By rotating of a plane parallel Al-plate through the divergent partial neutron beams after the dividing plate of the interferometer, an additional relative phase ∆*φ*_A1_ between the partial wave functions can be superimposed. The total phase shift is then the sum of this phase shifter contribution, a sample contribution ∆*φ*_sample_ and an additional, time-dependent instrumental intrinsic phase ∆*φ*_int_:

$$\Delta \varphi =\Delta \varphi_{A1}+\Delta \varphi_{\text{sample}}+\Delta \varphi_{\text{int}}.$$ (4)

**Fig. 1:**
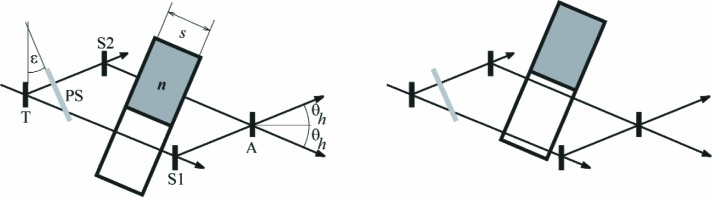
The partial beams emerging from the interferometer’s crystal plates (T, S1, S2, A) enclose the double Bragg angle 2*θ*_h_. In contrast, at the sample’s location the beams propagate nearly parallel. In the so-called sample position (left), the sample of refractive index *n* is placed in one partial beam, while the other partial beam passes through the evacuated compensation chamber. The twin cell is then moved to the so-called reference position, where both partial beams pass through the empty chamber (right). Now the phase shifter (PS) is rotated one angular step and one measures first in the reference position and then in the sample position. After a further rotation of (PS) the succession starts over until sample and reference measurements for 32 phase shifter positions ε have been performed.

Modulating the total phase by means of the phase shifter enables one to extract both modulus and sign of ∆*φ*_sample_ + ∆*φ*_int_. The intrinsic phase shift of the instrument can be monitored when the phase shift through an identical but evacuated sample cell is measured periodically. The skew symmetry of the instrument has the consequence that the partial beams are almost parallel in the volume where the sample is placed, see Fig. 1. Therefore, the aluminum windows of the gas container do not act as phase shifter and any misalignment of the container is compensated. Further, misalignment of the cell to better than 2.5° (which is easily achieved using a theodolite) results in an increase of the effective sample thickness of less than 10^−4^.

**Fig. 2:**
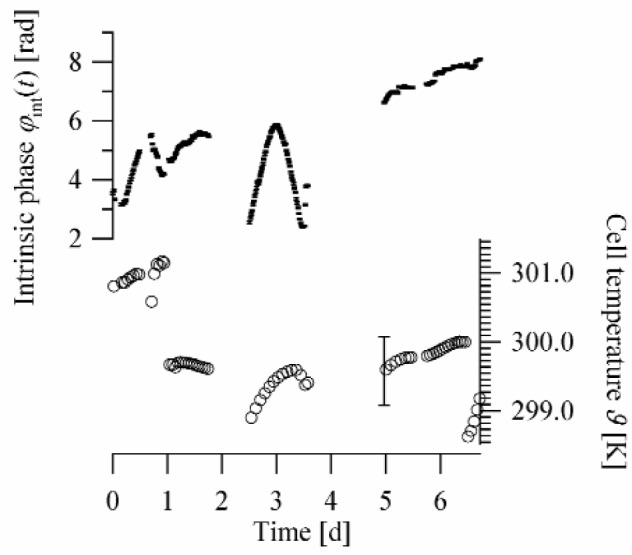
The intrinsic phase of the instrument is obviously temperature correlated. Phase drift rates up to $\dot{\varphi }=\pi /(12\;h)$ were observed.

The temperature correlated phase drifts of the instrument, c.f. Fig. 2, would lead to a systematic error in the phase shift if they were not compensated for. Simulation calculations suggest an asymmetrically alternating measuring sequence. By this we mean the following procedure: for a fixed phase shifter position, neutrons are counted in both cell positions, first in the sample and thereafter in the reference position, say. Then, without changing the cell position, the phase shifter is moved one step and held fixed again. The subsequent two measurements are then performed in opposite order, first when the cell is in the reference position and then in the sample position. After that, the phase shifter is rotated one step further and the measurements’ order starts over until data for 32 phase shifter positions are recorded. Performing alternating measurements with and without sample in changing succession for every phase shifter position guarantees that even phase drifts during some 10 s are compensated.

Moreover, the simulation calculation indicates that harmonic fits of the phase have to be carried out relative to the center of the data. In this case, the absolute difference between the real phase shift and the fit result can in principle be made arbitrarily small.

**Fig. 3:**
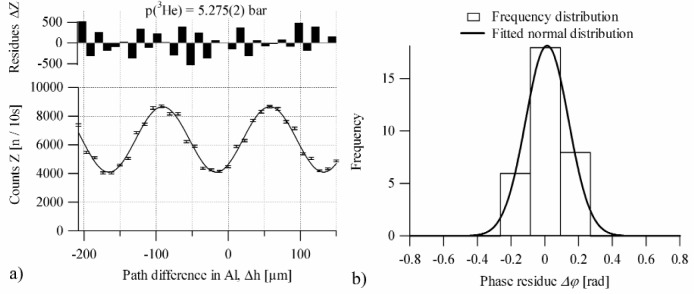
a) Raw data (bottom) and residuals of the individual data points (top). Since the residuals do not show any systematical pattern, the width of their frequency distribution b) can be used as an estimate for counting uncertainty contributions through integrating over fast varying phase fluctuations.

The data show deviations from the expected sine behavior, which cannot be accounted for by Poisson statistics, see Fig. 3a. Instead, the deviations are assumed to be the result of integrating over short-time phase fluctuations. In order to include this error contribution, counting residuals were transformed into phase residuals, and histogrammed as shown in Fig. 3b. Neither the distribution of phase residuals nor the histogrammed data show any repetitive pattern, thereby confirming the statistical nature of the underlying mechanism. Moreover, the standard deviation and the Gaussian width of the frequency distributions of the residuals are consistent in all cases. Typical phase fluctuations are of the order of some few tenths of radians. When this “phase fluctuation” contribution is combined with counting statistics, the data is well described by cosines. The reduced chi-squared values near 1 do not indicate any difficulties with this error analysis procedure.

Since the relative intensities of the partial beams were not constant during the experiment, transmission data could not be used to extract the gas particle number density from the known absorption cross section. Instead, the temperature and pressure of the sample were monitored using DKD [3] calibrated sensors, and the density was calculated from the state equation of real gases.

**Fig. 4:**
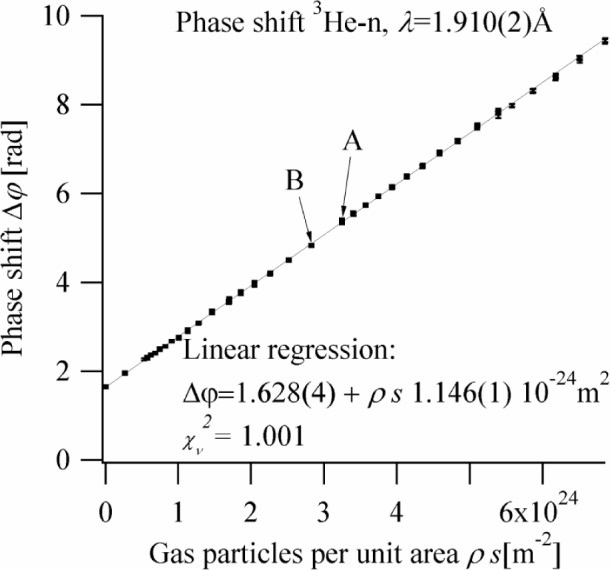
Observed phase shifts as a function of gas particle number density times sample thickness. The slope of the linear regression divided by the neutron wave length yields essentially the real part of coherent bound scattering length, $b_{c}^{\prime }[1-2kb_{c}^{\prime \prime }+O(k^{2})]$.

The neutron wavelength was determined to be λ = (1.910 ± 0.002) Å by measuring the angular difference between the Bragg peaks of the dispersive and nondispersive crystal orientations. Figure 4 shows the measured phase shifts of the individual measurements as a function of particle number area density. In accordance with Eqs. (1) and (2), the data show a strictly linear behaviour. Dividing the slope of the linear regression by the neutron wavelength and correcting for terms in $kb_{c}^{\prime \prime }$ yields our final result of $b_{c}^{\prime }=\left(6.000\pm 0.009\right)\;\text{fm}$.

We stress that there were over 13 h of measurement interruption combined with cell removal, soldering and cell replacement between the data points marked A and B. While one normally would avoid such “heavy work” during interferometric experiments, at the level of our precision the data taken after point B do not show a systematic shift compared to the data taken before point A. Thus, we believe that the indications of the simulation calculations are confirmed by experiment and that our phase drift compensation scheme indeed works very effectively.

## 3. Discussion

Compared to the currently accepted value of $b_{c}^{\prime }=\left(5.74\pm 0.07\right)\;\text{fm}$, that also was measured using neutron interferometry [4], our result shows a significant 4 σ deviation towards a higher value. This observation is confirmed by an independent experiment at NIST [5] using largely the same technique. Compared to the latest NIST result, there is a 12 σ discrepancy in the results, which cannot be resolved.

## Footnotes

1 Certain commercial equipment, instruments, or materials are identified in this paper to foster understanding. Such identification does not imply recommendation or endorsement by the National Institute of Standards and Technology, nor does it imply that the materials or equipment identified are necessarily the best available for the purpose.
